# Ultra-deep massively parallel sequencing with unique molecular identifier tagging achieves comparable performance to droplet digital PCR for detection and quantification of circulating tumor DNA from lung cancer patients

**DOI:** 10.1371/journal.pone.0226193

**Published:** 2019-12-16

**Authors:** Le Son Tran, Hong-Anh Thi Pham, Vu-Uyen Tran, Thanh-Truong Tran, Anh-Thu Huynh Dang, Dinh-Thong Le, Son-Lam Nguyen, Ngoc-Vu Nguyen, Trieu-Vu Nguyen, Binh Thanh Vo, Hong-Thuy Thi Dao, Nguyen Huu Nguyen, Tam Huu Tran, Chu Van Nguyen, Phuong Cam Pham, Anh Tuan Dang-Mai, Thien Kim Dinh-Nguyen, Van Hieu Phan, Thanh-Thuy Thi Do, Kiet Truong Dinh, Han Ngoc Do, Minh-Duy Phan, Hoa Giang, Hoai-Nghia Nguyen

**Affiliations:** 1 Gene Solutions, Ho Chi Minh, Vietnam; 2 Medical Genetics Institute, Ho Chi Minh City, Vietnam; 3 Graduate program of Genetics, Ho Chi Minh city University of Science, Ho Chi Minh city, Vietnam; 4 University of Medicine and Pharmacy, Ho Chi Minh city, Vietnam; 5 Pham Ngoc Thach Hospital, Ho Chi Minh city, Vietnam; 6 Thu Duc Hospital, Ho Chi Minh city, Vietnam; 7 Center for Standardization and QC in Medical Lab of Ho Chi Minh City, Ho Chi Minh City, Vietnam; 8 Vietnam National Cancer Hospital, Ha Noi, Vietnam; 9 Bach Mai Hospital, Ha Noi, Vietnam; 10 Center for Forensic Science, Ho Chi Minh City, Vietnam; 11 Tan Hung General Hospital, Ho Chi Minh City, Vietnam; 12 School of Chemistry and Molecular Biosciences, University of Queensland, Brisbane, Australia; University Hospital Modena and Reggio Emilia, ITALY

## Abstract

The identification and quantification of actionable mutations are of critical importance for effective genotype-directed therapies, prognosis and drug response monitoring in patients with non-small-cell lung cancer (NSCLC). Although tumor tissue biopsy remains the gold standard for diagnosis of NSCLC, the analysis of circulating tumor DNA (ctDNA) in plasma, known as liquid biopsy, has recently emerged as an alternative and noninvasive approach for exploring tumor genetic constitution. In this study, we developed a protocol for liquid biopsy using ultra-deep massively parallel sequencing (MPS) with unique molecular identifier tagging and evaluated its performance for the identification and quantification of tumor-derived mutations from plasma of patients with advanced NSCLC. Paired plasma and tumor tissue samples were used to evaluate mutation profiles detected by ultra-deep MPS, which showed 87.5% concordance. Cross-platform comparison with droplet digital PCR demonstrated comparable detection performance (91.4% concordance, Cohen’s kappa coefficient of 0.85 with 95% CI = 0.72–0.97) and great reliability in quantification of mutation allele frequency (Intraclass correlation coefficient of 0.96 with 95% CI = 0.90–0.98). Our results highlight the potential application of liquid biopsy using ultra-deep MPS as a routine assay in clinical practice for both detection and quantification of actionable mutation landscape in NSCLC patients.

## Introduction

Cancer of the lung is the leading type of cancer, responsible for the highest number of new cases and the largest number of deaths worldwide [1]. Non-small cell lung cancer (NSCLC) is the most common subtype, accounting for approximately 85% of all cases [2]. The majority of NSCLC patients display advanced disease when diagnosed and thus have poor prognosis [2, 3]. Treatment options for NSCLC patients are based on the stage of the cancer but high recurrence rate of 30–70% is expected after surgical resection [4]. In patients with advanced stage or tumor recurrence, the mutation profiles of cancer tissue are vital to guide targeted therapy and monitor the tumor recurrence, thereby improving the survival rate of advanced NSCLC patients [4, 5].

Acquired genetic alterations in the *EGFR*, *KRAS*, *NRAS*, *BRAF*, *ROS1* and *ALK* oncogenes are the most common mutations in NSCLC and certain mutations are associated with drug sensitivity or resistance [6, 7]. Advanced NSCLC patients harbouring activating *EGFR* mutations including deletion in exon 19 (del19) or a point mutation L858R in exon 21 (L858R) exhibited longer progressive-free survival after receiving treatment with gefitinib, a tyrosine kinase inhibitor (TKI) [8–10]. However, patients treated with the first and second generation TKI drugs such as afatinib and gefitinib often develop a TKI resistant mutation T790M in *EGFR* exon 20 after a median period of 12 months [11, 12]. In such cases, a third generation TKI drug, osimertinib, has been shown to be effective against cells with the T790M mutation [13]. Apart from mutations in *EGFR*, a significant proportion of NSCLC patients harbour somatic mutations in other oncogenes, downstream effector molecules of the EGFR pathway, including *KRAS* (15–25%), *BRAF* (1–3%) and *NRAS* (1%) [14, 15]. It has been reported that carriers of *NRAS* and *BRAF* mutations display distinct clinicopathologic features and that *BRAF* mutation testing has recently been recommended for NSCLC patients by American Society of Clinical Oncology (ASCO) [14, 16, 17]. Patients with *KRAS* mutations were shown to develop resistance to the current EGFR targeted therapies, supporting the use of *KRAS* mutations as negative prediction biomarkers [18]. However, its clinical significance has been challenged by recent meta-analysis studies reporting inconsistent results amongst different patient cohorts [19–21]. Nevertheless, these studies highlighted that comprehensive mutation analysis of cancer driver genes is essential to provide NSCLC patients with the optimal treatment regimen.

Tumor tissue biopsy is regarded as the gold standard for tumor genetic profiling in current clinical practice [22]. However, since this is an invasive procedure, it is not always feasible to carry out the biopsy to assess patients’ responses following initial treatment, particularly in those who are in advanced stages or do not have sufficient tumor tissues [23]. Liquid biopsy has recently been shown to better reflect the whole genetic complexity of tumor tissues and enables real-time monitoring of treatment-associated resistance [24, 25]. This approach involves detecting genetic alterations in circulating tumor DNAs (ctDNA), which are 160–200 bp DNA fragments released into the blood circulation by tumor cells undergoing cell death [24]. However, the low abundance of ctDNA as well as low variant allele frequency (VAF) of somatic mutations in human plasma necessitates the use of a highly sensitive analytical technique for genetic assessment in liquid biopsy [26]. Several methods have been developed to detect low VAF mutations in plasma, including targeted methods such as amplification refractory mutation system (ARMS) and droplet digital PCR (ddPCR) or non-targeted genome wide massively parallel sequencing (MPS) [27–30]. However, both ARMS and MPS are not sensitive enough to detect low VAF mutations in plasma samples, discouraging its application in liquid biopsy [29, 31, 32]. In contrast, ddPCR has been shown to achieve high sensitivity and accuracy for both identification and quantification of mutations in ctDNA, enabling the evaluation of the intra-tumor progression of drug sensitive or resistant mutant clones [27, 33]. However, this technology relies on prior knowledge of tumor genetic constitution and only allows analyzing a limited number of mutations per reaction.

Recent advances in MPS technology such as unique molecular barcoding has made substantial improvements on its sensitivity and accuracy [34–37]. Unlike ddPCR, MPS is capable of exploring the complete mutation landscape of multiple driver genes simultaneously [38]. This provides particular advantage for longitudinal monitoring of tumor progression and recurrence following initial treatments, and could lead to the discovery of novel mutations that might be of clinical significance [38]. With enhanced sensitivity and accuracy, we believe ultra-deep MPS with unique molecular identifier tagging represents a promising method applicable for liquid biopsy.

In the present study, we adopted ultra-deep MPS for liquid biopsy and evaluated its clinical use for both detection and quantification of plasma circulating tumor DNA in advanced NSCLC patients. The performance of ultra-deep MPS was also compared against that of ddPCR to demonstrate a comparable performance with added benefit of detecting more mutations in more target genes.

## Methods

### Patient recruitment

**Fig 1:**
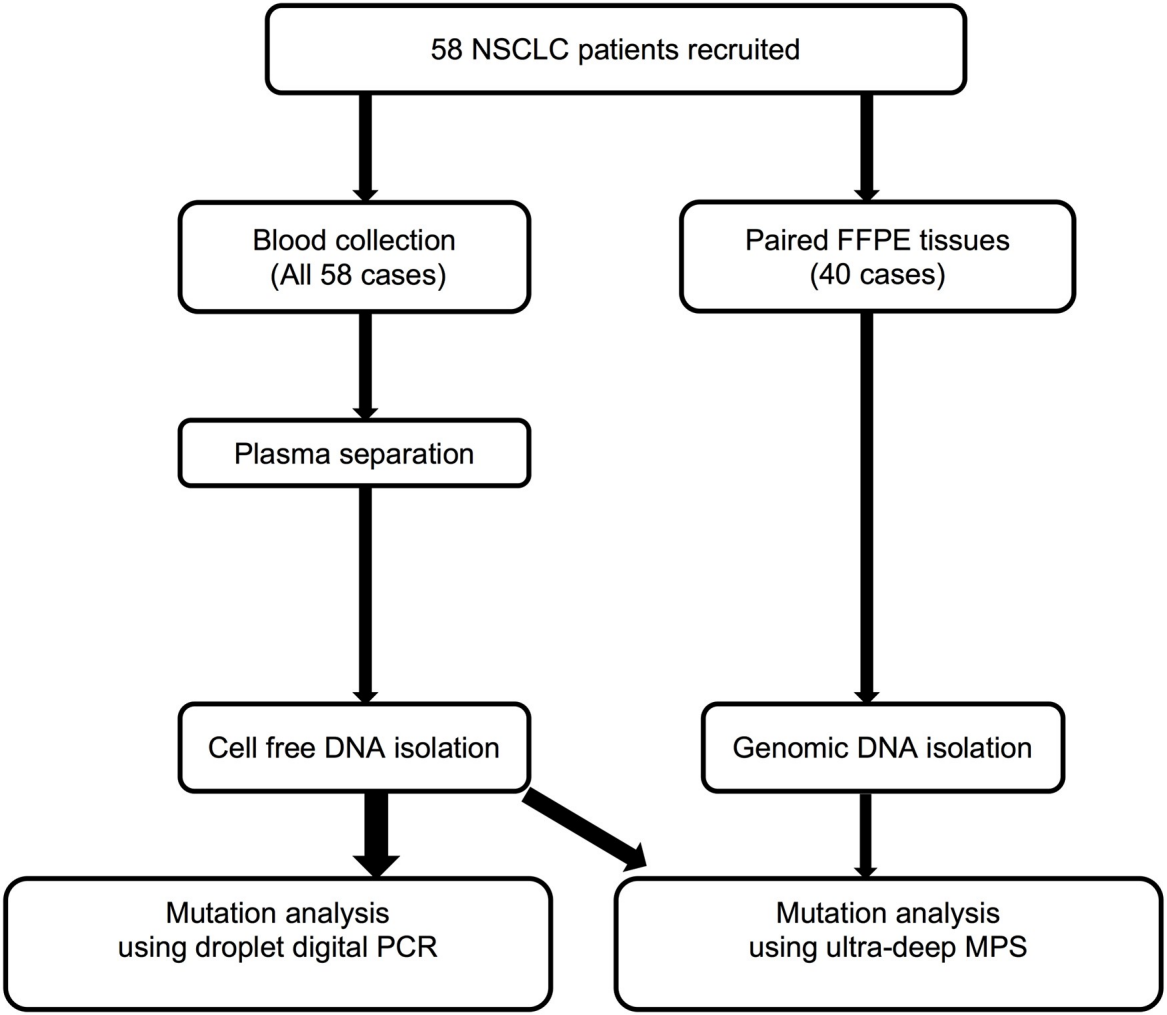
Schematic diagram of the sample handling procedure.

A total of 58 patients diagnosed with NSCLC from Pham Ngoc Thach hospital, Thu Duc district hospital, Ho Chi Minh City and National cancer hospital Vietnam were recruited to this study, 40 of which provided paired samples of tissue biopsies and plasma, while the remaining 18 provided only plasma samples (Fig 1). Written informed consents were obtained from all patients. Comprehensive details of patients’ clinical factors were summarised in S1 Table and listed in S2 Table. This study was approved by The Ethic Committee of University of Medicine and Pharmacy at Ho Chi Minh City, Vietnam (Ethic number: 027/DHYD-HD). Case No. 50, 51, 52 and 53 were confirmed to experience TKI treatment.

### Clinical sample collection

Prior to tissue biopsy, 10 mL of peripheral blood was drawn in K2-EDTA tubes (BD Vacutainer, USA), stored at room temperature for maximum of 8 hours before 2 rounds of centrifugation (2,000 x g for 10 min then 16,000 x g for 10 min) to separate plasma from blood cells. The plasma (4–6 mL) was then collected, aliquoted (2 mL per aliquot) and stored at -80°C until cell free DNA extraction. Tissue biopsies were collected, formalin-fixed and paraffin-embedded (FFPE) and then the tumor-rich areas of the FFPE tissues that contain at least 50% of tumor cells identified by a hematoxylin and eosin staining were micro-dissected.

### DNA isolation

Cell free DNA was extracted from an aliquot of 2 mL of plasma using the MagMAX Cell-Free DNA Isolation kit (Thermo Fisher, USA) following the manufacturer’s instructions. Tumor tissue-derived DNA was extracted from FFPE samples using QIAamp DNA FFPE Tissue Kit (Qiagen, USA) following the manufacturer’s instructions. Both cell free DNA from plasma and genomic DNA from FFPE (2μl of sample) were then quantified using the QuantiFluor dsDNA system (Promega, USA) and Quantus Fluorometer (Promega, USA).

### Ultra-deep massively parallel sequencing with unique molecular identifier tagging

For cell free DNA (cfDNA), library with unique molecular identifier tagging were prepared from 2 ng of cfDNA using the Accel-NGS 2S Plus DNA library kit (Swift Biosciences, USA) following the manufacturer’s instructions. Library concentrations were quantified with a QuantiFluor dsDNA system (Promega, USA). Equal amounts of libraries were pooled together and hybridized with xGen Lockdown probes for four targeted genes *EGFR*, *KRAS*, *NRAS*, *BRAF* (IDT DNA, USA). Sequencing was performed using NextSeq 500/550 High output kits v2 (150 cycles) on Illumina NextSeq 550 system (Illumina, USA) with the coverage of 10.000X.

For genomic DNA from FFPE, libraries were prepared from 2 ng of cfDNA using the NEBNext Ultra II FS DNA library prep kit (New England Biolabs, USA) following the manufacturer’s instructions. Similar to ctDNA libraries, FFPE libraries were pooled before hybridization with the xGen Lockdown probes and sequencing in the Illumina NextSeq 550 system. Both cfDNA and FFPE samples exhibited 5–10% on-target reads.

### Variant calling using Mutect2

For ctDNA, each sample was barcoded with a single 8-bp index in the P7 primer and each DNA fragment were tagged with a unique identifier consisting of a random 9-bp sequence within the P5 primer. Pair-end (PE) reads and the correspondent unique identifier sequences were generated using bcl2fastq package (Illumina). The reads were aligned to human genome (hg38) using BWA package and then grouped by the unique identifier in order to determine a consensus sequence for each fragment, eliminating sequencing and PCR errors that account for less than 50% of reads per fragment. The consensus reads were used for final variant calling using Mutect2. A custom pipeline with call to BWA, Picard, Samtools and Fulcrum genomic analysis packages were built to perform the above-mentioned analysis steps.

For genomic DNA from FFPE samples, each sample was barcoded with dual indexes in the P7 and P5 primer. The PE reads were generated by bcl2fastq package (Illumina) and aligned to human genome (hg38) using BWA package. Duplicate reads were marked using MarkDuplicates from Picard tools (Broad Institute). Somatic variants were called using Mutect2 package (Broad Institute). A custom pipeline with call to BWA, Picard, and Samtools packages were built to perform the above-mentioned analysis steps.

### ddPCR method

A four-step ddPCR procedure was performed using reagents and equipment from Bio-Rad (unless otherwise stated) following the manufacturer’s instruction [39]. Briefly, the PCR mix was first prepared by mixing 1 × ddPCR Supermix for Probes, primers and probes (IDT DNA) and DNA template (0.8 or 1.6 ng). Next, 20 μl of the PCR mix was transferred into the Droplet Generator DG8TM Cartridge followed by 70 μl of the Droplet Generation Oil before placing in a QX100TM Droplet Generator to generate droplets. Subsequently, the droplets were transferred to a 96-well plate before placing in a thermal cycler (C1000 Touch, Bio-Rad) for PCR amplification. The PCR thermal program was performed as follows: 95°C for 10 min, then 40 successive cycles of amplification (94°C for 30 sec; 55°C for 60 sec) and 98°C for 10 min. Lastly, the droplet reading was acquired by the QX 200 Droplet reader and analyzed using the QuantaSoft Software. Positive and negative droplets were assigned based on the fluorescence threshold that was set as previously described by Deprez *et al*. [40].

To detect T790M and L858R mutations in exon 20 and 21 of the *EGFR* gene, one reaction of ddPCR was used with two sets of primers and probes as follows: T790M primer F-GCCTGCTGGGCATCTG; T790M primer R-TCTTTGTGTTCCCGGACATAGTC; T790M mutation probe FAM- ATGAGCTGCATGATGAG-ZEN/3'IBFQ; L858R primer F-GCAGCATGTCAAGATCACAGATT; L858R primer R-CCTCCTTCTGCATGGTATTCTTTCT; L858R mutation probe HEX-AGTTTGGCCCGCCCAA- ZEN/3'IBFQ. For detection of 15 deletion sites in exon 19 (del19) of the *EGFR* gene, a commercially available ddPCR reaction (Bio-rad) was used (ddPCR^™^ EGFR Exon 19 Deletions Screening Kit #12002392).

### Determination of limit of detection

To determine the limit of detection (LOD) for our assays, we first performed fragmentation of reference wild type (WT) and mutant DNA (Tru-Q1 and Tru-Q2, Horizon) to create 100–200 bp fragments corresponding to the general length of plasma cell-free DNA. Subsequently, these mutant DNA fragments were spiked into fragmented WT DNA to obtain a series of standard samples with a desired variant allele frequency (VAF) range. The LOD value was defined as the lowest VAF that can be reliably detected by ddPCR or Ultra deep MPS. The LOD values of ddPCR and ultra-deep MPS assay for detecting major driver mutations in plasma were 0.5% and 1%, equivalent to 5 and 10 mutant copies per 1,000 copies of DNA input, respectively.

### Statistical analysis

All statistical tests and visualisation plots were performed using R, the ggplot2 and ggpubr packages. Cohen’s Kappa coefficient and its confidence intervals using the psych package were employed to assess the reliability of mutation detection by ddPCR and MPS. Pearson’s correlation coefficient and Bland-Altman’s plot were performed to examine the correlations and agreement, respectively, between VAF results obtained by the two methods. To assess the reliability of VAF quantification, Intraclass correlation coefficient (ICC) estimates and their 95% confident intervals were calculated using irr package based on single rater type, consistency definition, and a 2-way random-effects model.

## Results

### High concordance between mutations detected by paired liquid and tissue biopsy

In this study, we developed a liquid biopsy protocol based on ultra-deep Illumina sequencing with unique molecular identifier tagging for detecting mutations in four genes *EGFR*, *KRAS*, *NRAS* and *BRAF* for patients with advanced NSCLC. To evaluate the mutations detected by liquid biopsy, we examined the concordance between mutations detected from plasma samples and from tissue samples in the cohort of 40 patients who provided paired plasma-tissue samples (Table 1). Within this cohort, liquid biopsy detected 9 types of mutations in two genes *EGFR* and *KRAS*, while no mutation was detected in either *BRAF* or *NRAS* gene (Table 1). Deletions in exon 19 of *EGFR* (del19) were the most common, found in 5 plasma samples (Table 1, Case No. 4–8), followed by L858R in *EGFR* in 3 samples (Table 1, Case No. 1–3), then insertion in *EGFR* exon 20 in 2 samples (Table 1, Case No. 14 and 35). The remaining mutations were found in one sample each, including H773A, S768C, in *EGFR* (Table 1, Case No. 15 and 21) and G12V, G12C and G12D in *KRAS* (Table 1, Case No. 30, 32 and 37). One plasma sample was found to harbour double mutations of L858R and T790M, a TKI resistant mutation, in *EGFR* (Table 1, Case No. 11). Compared to the mutations detected in tissue samples, the assays showed high concordance rate of 87.5% (35/40), including 15 patients with matching mutation profiles and 20 patients with no mutation detected (Table 1). Among the five discordant cases, four were positive for mutations in *EGFR* in tissue but negative in plasma (Table 1, Case No. 10,12, 13 and 24), while one was negative in tissue but positive for *EGFR* del19 in plasma (Table 1, Case No. 5).

**Table 1:** Mutation results of 40 plasma and matched tumor tissue samples detected by ultra-deep MPS.

Case No.	Sample ID	NGS Results			
		Plasma		Tumor tissues	
		Mutation	VAF (%)	Mutation	VAF (%)
1	LBL015	*EGFR* L858R	89	*EGFR* L858R	65
2	LBL017	*EGFR* L858R	2	*EGFR* L858R	90
3	L10055	*EGFR* L858R	1	*EGFR* L858R	55
4	L10019	*EGFR* del19	17	*EGFR* del19	11
5	L10021	*EGFR* del19	6	(-)	
6	L10036	*EGFR* del19	50	*EGFR* del19	44
7	L10072	*EGFR* del19	8	*EGFR* del19	50
8	L10076	*EGFR* del19	8	*EGFR* del19	34
9	LBL021	(-)		(-)	
10	LBL033	(-)		*EGFR* del19	20
11	L10022	*EGFR* L858R	1	*EGFR* L858R	37
		*EGFR* T790M	5	*EGFR* T790M	43
12	LBL026	(-)		*EGFR* del19	23
13	LBL030	(-)		*EGFR* del19	10
14	LBL001	*EGFR* ins20	1.5	*EGFR* ins20	25
15	LBL002	*EGFR* H773A	15	*EGFR* H773A	1
16	LBL003	(-)		(-)	
17	LBL004	(-)		(-)	
18	LBL005	(-)		(-)	
19	LBL006	(-)		(-)	
20	LBL007	(-)		(-)	
21	LBL008	*EGFR* S768C	1.5	*EGFR* S768C	45
22	LBL009	(-)		(-)	
23	LBL012	(-)		(-)	
24	LBL013	(-)		*EGFR* ins20	20
25	LBL014	(-)		(-)	
26	LBL016	(-)		(-)	
27	LBL020	(-)		(-)	
28	LBL022	(-)		(-)	
29	LBL023	(-)		(-)	
30	LBL024	*KRAS* G12V	5	*KRAS* G12V	1
31	LBL025	(-)		(-)	
32	LBL027	*KRAS G12C*	3.5	*KRAS G12C*	23
33	LBL028	(-)		(-)	
34	LBL029	(-)		(-)	
35	LBL031	*EGFR* ins20	2.9	*EGFR* ins20	25
36	LBL034	(-)		(-)	
37	LBL036	*KRAS G12D*	1	*KRAS G12D*	1
38	LBL037	(-)		(-)	
39	LBL040	(-)		(-)	
40	LBL041	(-)		(-)	

(-): negative for tested mutation

### Comparable performance between ultra-deep MPS and droplet digital PCR (ddPCR) for *EGFR* mutation detection in plasma samples

Droplet digital PCR (ddPCR) has been reported to achieve high sensitivity and specificity for the detection of low frequency mutations such as those in ctDNA from plasma, with a limit of detection of less than 0.001% (1 copy of mutant DNA per 100,000 copies of wild-type DNA background) [26]. Using a commercially available ddPCR (Bio-rad) assay as a reference standard, we conducted a cross-platform comparison with ultra-deep MPS for the detection of the three most common actionable *EGFR* mutations (del19, L858R and T790M) in 58 plasma samples comprising the 40 previously tested samples and 18 additional samples (Table 2). The concordant rate between the two methods were 91.4% (53/58 samples), whereby for 19 cases, both methods agreed upon the mutation identified and 35 more cases were also congruent, by not having any of the 3 target mutations (Table 3).

**Table 2:** Mutational profile and variant allele frequency (VAF) determined by ultra-deep MPS and ddPCR in 58 plasma samples.

Case No.	Sample ID	Ultra-deep MPS Results		ddPCR Results	
		Mutation	VAF (%)	Mutation	VAF (%)
1	LBL015	*EGFR* L858R	89	*EGFR* L858R	86.0
2	LBL017	*EGFR* L858R	2	*EGFR* L858R	2.8
3	L10055	*EGFR* L858R	1	*EGFR* L858R	4.6
4	L10019	*EGFR* del19	17	*EGFR* del19	28.6
5	L10021	*EGFR* del19	6	*EGFR* del19	7.0
6	L10036	*EGFR* del19	50	*EGFR* del19	68.4
7	L10072	*EGFR* del19	8	*EGFR* del19	12.8
8	L10076	*EGFR* del19	8	*EGFR* del19	9.5
9	LBL021	(-)		*EGFR* del19	0.7
10	LBL033	(-)		*EGFR* del19	0.5
11	L10022	*EGFR* L858R	1	*EGFR* L858R	0.75
		*EGFR* T790M	5	*EGFR* T790M	0.75
12	LBL026	(-)		(-)	
13	LBL030	(-)		(-)	
14	LBL001	*EGFR* ins20	1.5	NA	
15	LBL002	*EGFR* H773A	15	NA	
16	LBL003	(-)		(-)	
17	LBL004	(-)		(-)	
18	LBL005	(-)		(-)	
19	LBL006	(-)		(-)	
20	LBL007	(-)		(-)	
21	LBL008	*EGFR* S768C	1.5	NA	
22	LBL009	(-)		(-)	
23	LBL012	(-)		(-)	
24	LBL013	(-)		(-)	
25	LBL014	(-)		(-)	
26	LBL016	(-)		(-)	
27	LBL020	(-)		(-)	
28	LBL022	(-)		(-)	
29	LBL023	(-)		(-)	
30	LBL024	*KRAS* G12V	5	NA	
31	LBL025	(-)		(-)	
32	LBL027	*KRAS G12C*	3.5	NA	
33	LBL028	(-)		(-)	
34	LBL029	(-)		(-)	
35	LBL031	*EGFR* ins20	2.9	NA	
36	LBL034	(-)		(-)	
37	LBL036	*KRAS G12D*	1	NA	
38	LBL037	(-)		(-)	
39	LBL040	(-)		(-)	
40	LBL041	(-)		(-)	
41	LBL019	*EGFR* L858R	0.8 *	*EGFR* L858R	1.2
42	LBL032	(-)		*EGFR* L858R	0.9
43	LBL035	*EGFR* L858R	0.7 *	*EGFR* L858R	2.8
44	L10002	*EGFR* L858R	3	*EGFR* L858R	5.8
45	L10045	*EGFR* L858R	20	*EGFR* L858R	15.9
46	L10077	*EGFR* L858R	8	*EGFR* L858R	6.1
47	LBL038	(-)		*EGFR* del19	3.4
48	LBL010	*EGFR* del19	5	*EGFR* del19	12.1
49	L10043	*EGFR* del19	23	*EGFR* del19	36.0
50	L10005	*EGFR* L858R	4	*EGFR* T790M	1.5
				*EGFR* L858R	3.9
51	L10046	*EGFR* L858R	24	*EGFR* T790M	18
		*EGFR* T790M	56	*EGFR* L858R	61.7
52	L10007	*EGFR* T790M	16	*EGFR* T790M	12.9
		*EGFR* del19	20	*EGFR* del19	34
53	L10074	*EGFR* T790M	15	*EGFR* T790M	15
		*EGFR* del19	31	*EGFR* del19	37.7
54	LBL011	(-)		(-)	
55	LBL018	(-)		(-)	
56	LBL039	(-)		(-)	
57	LBL042	(-)		(-)	
58	LBL043	(-)		(-)	

(-): negative for tested mutations; NA: mutations not analysed by ddPCR;

(*): VAF below the limiting detection of Ultra-deep MPS

**Table 3:** Evaluation of performance of ddPCR and MPS for mutation detection in 58 plasma samples.

NGS vs ddPCR	NGS			Performance results	
ddPCR	Mutation	Wild type	Total		
Mutation	19	5	24	Sensitivity	79.2%
Wild type	0	34	34	Specificity	100.0%
Total	19	39	58	Concordance	91.4%

Among the 5 discordant cases, 4 were negative by ultra-deep MPS while positive for del19 (Table 2, Case No. 9, 10 and 47) or L858R (Table 2, Case No. 42) by ddPCR; and 1 were a double mutation (L858R & T790M) by ddPCR but a single L858R mutation by ultra-deep MPS (Table 2, case No. 50). There were 7 cases where ultra-deep MPS detected mutations other than the three types targeted by ddPCR, illustrating an advantage of MPS over ddPCR (Table 2, case No. 14, 15, 21, 30, 32, 35 and 37).

If we considered ddPCR as a reference method and counted the 7 samples with mutations outside of ddPCR detectable mutations as wild type, the sensitivity and specificity of the ultra-deep MPS assay for *EGFR* mutation detection in plasma samples were 79.2% (19/24, 95% CI = 57.8%-92.9%) and 100% (34/34), respectively, with an accuracy of 91.4% (53/58) (Table 3). The Cohen’s kappa coefficient was 0.85 (95% CI = 0.72–0.97), suggesting good agreement between the two methods. Taken together, these results demonstrated that liquid biopsy using ultra-deep MPS achieved good agreement with ddPCR for the detection of mutations from ctDNA in plasma samples.

### Quantitative measurement of mutation allelic frequency by ultra-deep sequencing and ddPCR

**Fig 2:**
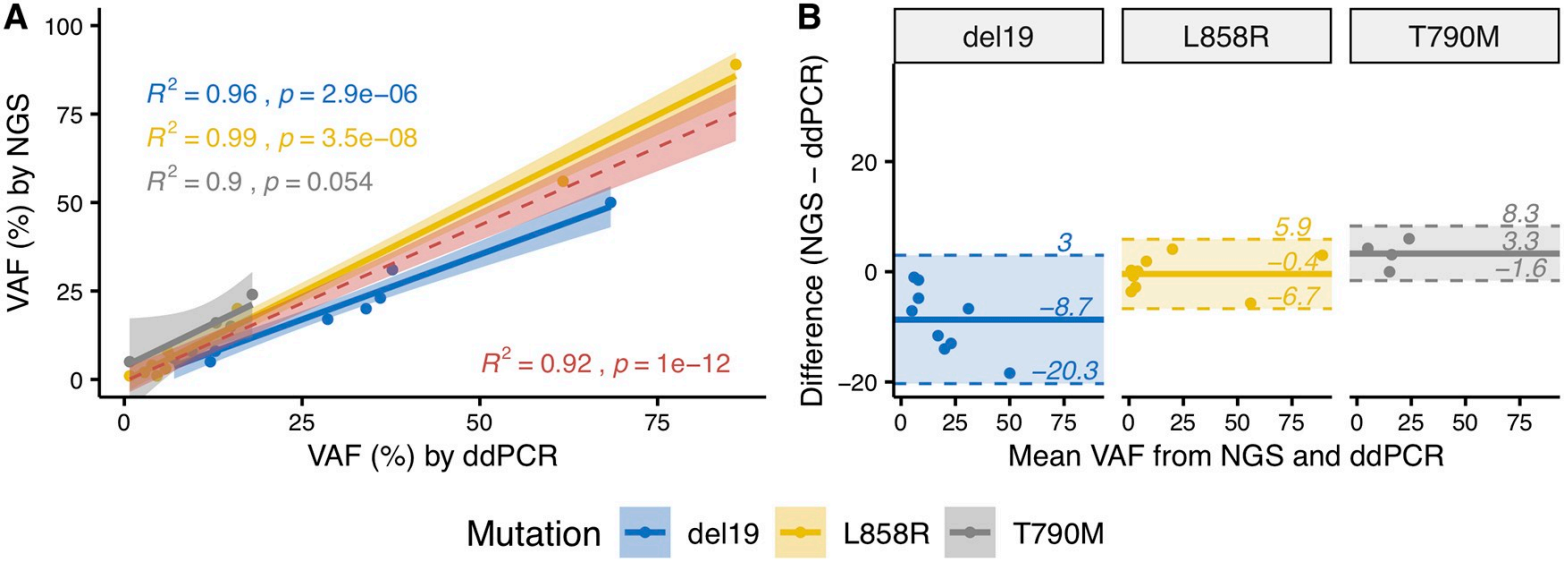
Comparing mutation allele frequency quantified from plasma by ultra-deep MPS and ddPCR. (A) Linear regression and Pearson’s correlation coefficients of VAFs in plasma samples as determined by ddPCR and ultra-deep MPS with unique molecular identifier tagging. VAFs of del19, L858R and T790M mutations in *EGFR* were analysed separately (blue, yellow and grey, respectively) and combined (red) to show that MPS achieved significant correlation with ddPCR. (B) Bland-Altman plots demonstrating the agreement between ultra-deep MPS and ddPCR in quantifying VAFs of the three mutation types in *EGFR* from plasma samples.

Besides high mutation detection sensitivity in ctDNA, ddPCR also shows the capability of absolute mutation quantification, allowing better disease prognosis and therapy response monitoring [26, 41]. To evaluate the quantitative measurement of VAF by ultra-deep sequencing, we compared the VAF for the three *EGFR* mutations (del19, L858R and T790M) with those reported by ddPCR. VAFs reported by the two methods exhibited a strong overall Pearson’s linear correlation (R^2^ = 0.92, *P* <0.0001) (Fig 2A). More specifically, VAFs of L858R mutation showed the best correlation (R^2^ = 0.99, *P* <0.0001), followed by VAFs of del19 (R^2^ = 0.96, *P* <0.0001), then by VAFs of T790M (R^2^ = 0.90, *P* = 0.05) (Fig 2A). Intraclass correlation coefficient (ICC) for the two methods was estimated at 0.96 (95% CI = 0.90–0.98), indicates excellent reliability. Bland-Altman analysis revealed relatively high level of agreement between two methods, of which del19 mutations showed the largest range of limits of agreement (LOA) from -20.3% to 3%, followed by L858R from -6.7% to 5.9% and T790M from -1.6 to 8.3% (Fig 2B). Thus, the liquid biopsy based on ultra-deep sequencing exhibited comparable quantitative measurement of VAF to that of ddPCR.

## Discussion

The American Society of Clinical Oncology (ASCO) has stated that the identification of somatic driver mutations is essential for designing optimal treatment regimens for NSCLC patients [17]. The technical and clinical limitations of traditional tissue biopsy necessitate the development of liquid biopsy, a procedure of detecting and quantifying tumor-derived mutations from ctDNA found in plasma samples of cancer patients [23, 24]. The choice of analytical platform for liquid biopsy requires proper evaluation, taking into account sensitivity, repeatability, discoverability and feasibility in clinical settings [42, 43].

In this study, we aimed to demonstrate that ultra-deep MPS with unique molecular identifier tagging is suitable for liquid biopsy to detect and quantify mutations in ctDNA of NSCLC patients. First, we used paired plasma and tumor tissue samples to examine whether liquid biopsy using ultra-deep MPS could detect the mutation profiles found in tumor tissues. Despite of the small sample size of this cohort (n = 40), the mutation profiles identified support previous findings that the majority of adenocarcinomas associated mutations occur in *EGFR* exon 19 and 21 and that *KRAS* and *EGFR* mutations are mutually exclusive [44–46]. Although *NRAS* and *BRAF* mutations have been found in NSCLC patients, none of the cases in this cohort was identified to carry such mutations. At high concordance rate of 87.5% between liquid and tissue biopsies, our results indicated that ultra-deep MPS could be useful for exploring the mutational landscape of NSCLC in clinical practice. There were four cases where mutations in *EGFR* (3 del19 and 1 ins20) were found in tissue but not in paired plasma samples, probably due to the low abundance of ctDNA in plasma [47]. Indeed, assaying these three plasma samples (del19 in tissue) by ddPCR showed that two were also negative and 1 with low VAF of 0.5% (Table 1, Case No. 10, 12 and 13). In contrast, there was one case where *EGFR* del19 mutation was detected in plasma but not in its paired tissue. This could be explained by the intratumoral genetic heterogeneity with the presence multiple cancer clones [47]. To address these issues, the current ASCO guidelines recommend that positive testing results in plasma would allow drawing definitive conclusion about the presence of mutation and that wild-type testing results in liquid biopsy be retested using tissue biopsy [42].

Second, by using ddPCR targeting three clinically actionable mutations in *EGFR* (del19, L858R and T790M) as the reference method, we conducted a cross-platform comparison of the performance of ultra-deep MPS in detecting these three mutations in 58 plasma samples. Ultra-deep MPS exhibited excellent concordance with ddPCR (91.4%), including 4/5 cases of double mutations (del19&T790M and L858R&T790M) (Table 2). The presence of T790M mutation in these patients was consistent with their previous treatment with first generation TKIs, suggesting that they might benefit from a third generation TKI therapy [41]. Ultra-deep MPS achieved sensitivity and specificity of 79.2% (19/24, 95% CI = 57.8%-92.9%) and 100% (34/34), respectively (Table 2). Of note, there were 5 cases positive by ddPCR but negative by ultra-deep MPS; three of which had VAF values lower than the LOD of the ultra-deep MPS assay (1%) while the other two also had low VAF (1.5% and 3.4%). Among those five cases, three did not have matched tissues to confirm ddPCR results (case No. 42, 47 and 50); one case was confirmed to have the mutation (del19) identified by ddPCR in matched tissue (case No. 10) and one case did not show any detectable mutation in matched tissue (case No. 9). Our data was consistent with previous studies reporting sensitivity value ranging from 70 to 80% for mutation detection in plasma of advanced NSCLC patients [48–50]. The Cohen’s kappa coefficient was 0.85 (95% CI = 0.72–0.97), further confirmed that ultra-deep MPS is comparable to ddPCR for the detection of 3 actionable mutations in *EGFR*. In additions, ultra-deep MPS showed extra advantage of ddPCR, capable of detecting more mutations than the limited set in ddPCR assays (Table 2).

Third, we investigated the ability of ultra-deep MPS with unique identifier tagging to quantify VAF in plasma samples. It has been reported that the relative abundance of activating and resistant mutations in *EGFR* is associated with patient survival rate and that the dynamic and quantitative analysis of *EGFR* mutations could guide personalized interventions [51]. Here, we demonstrated that ultra-deep MPS achieved accurate measurement of VAF values, showing great agreement with ddPCR (ICC = 0.96 with 95% CI = 0.90–0.98). However, the levels of agreement varied among the three mutations. Bland-Altman analysis (Fig 2B) showed that the LOA range is broadest for del19 mutation and ultra-deep MPS was more likely to give lower VAF estimates for del19 compared to those by ddPCR.

There were some limitations in our study. We could not calculate the costs of running ultra-deep MPS versus ddPCR in clinical settings. However, the reagent cost of a 4-gene panel using ultra-deep MPS was approximately that of ddPCR assays to detect two genetic alterations. Not all mutations detected by ultra-deep MPS were validated by ddPCR due to the limited number of assayed mutations in ddPCR. Although *ALK* and *ROS1* are clinically actionable genes in NSCLC, we did not include them in our ultra-deep MPS analysis because the genetic alterations frequently occur in these genes are rearrangement. Future work is required to solve the challenge of detecting gene rearrangements from ctDNA.

In conclusions, we have demonstrated that, in the context of liquid biopsy, our ultra-deep MPS with unique molecular identifier tagging achieved comparable performance to ddPCR for both the detection and quantification of clinically actionable mutations on plasma ctDNA. Altogether, our results highlight the potential application of liquid biopsy using modified MPS as a routine assay in clinical practice for both detection and quantification of actionable mutation landscape in NSCLC patients.

## Supporting information

S1 Table Clinical characteristics of NSCLC patients (n = 58) enrolled in this study. (XLSX)

S2 Table Summary of 58 patients’ clinical characteristics. (XLSX)
